# Plasma and memory antibody responses to Gamma SARS-CoV-2 provide limited cross-protection to other variants

**DOI:** 10.1084/jem.20220367

**Published:** 2022-07-07

**Authors:** Marianna Agudelo, Frauke Muecksch, Dennis Schaefer-Babajew, Alice Cho, Justin DaSilva, Eva Bednarski, Victor Ramos, Thiago Y. Oliveira, Melissa Cipolla, Anna Gazumyan, Shuai Zong, Danielle A.S. Rodrigues, Guilherme S. Lira, Luciana Conde, Renato Santana Aguiar, Orlando C. Ferreira, Amilcar Tanuri, Katia C. Affonso, Rafael M. Galliez, Terezinha Marta Pereira Pinto Castineiras, Juliana Echevarria-Lima, Marcelo Torres Bozza, Andre M. Vale, Paul D. Bieniasz, Theodora Hatziioannou, Michel C. Nussenzweig

**Affiliations:** 1 Laboratory of Molecular Immunology, The Rockefeller University, New York, NY; 2 Laboratory of Retrovirology, The Rockefeller University, New York, NY; 3 Howard Hughes Medical Institute, The Rockefeller University, New York, NY; 4 Laboratório de Biologia de Linfócitos, Programa de Imunobiologia, Instituto de Biofísica Carlos Chagas Filho, Universidade Federal do Rio de Janeiro, Rio de Janeiro, Brazil; 5 Departamento de Imunologia, Instituto de Microbiologia Paulo de Goes, Universidade Federal do Rio de Janeiro, Rio de Janeiro, Brazil; 6 Departamento de Doenças Infecciosas e Parasitárias, Faculdade de Medicina, Universidade Federal do Rio de Janeiro, Rio de Janeiro, Brazil; 7 Departamento de Genética, Ecologia e Evolução, Insituto de Ciências Biológicas, Universidade Federal de Minas Gerais, Belo Horizonte, Minas Gerais, Brazil; 8 Laboratório de Virologia Molecular, Instituto de Biologia, Universidade Federal do Rio de Janeiro, Rio de Janeiro, Brazil; 9 Núcleo de Vigilância Hospitalar, Hospital Federal do Andaraí, Ministério de Saúde, Rio de Janeiro, Brazil

## Abstract

Severe acute respiratory syndrome coronavirus 2 (SARS-CoV-2) continues to be a global problem in part because of the emergence of variants of concern that evade neutralization by antibodies elicited by prior infection or vaccination. Here we report on human neutralizing antibody and memory responses to the Gamma variant in a cohort of hospitalized individuals. Plasma from infected individuals potently neutralized viruses pseudotyped with Gamma SARS-CoV-2 spike protein, but neutralizing activity against Wuhan-Hu-1-1, Beta, Delta, or Omicron was significantly lower. Monoclonal antibodies from memory B cells also neutralized Gamma and Beta pseudoviruses more effectively than Wuhan-Hu-1. 69% and 34% of Gamma-neutralizing antibodies failed to neutralize Delta or Wuhan-Hu-1. Although Class 1 and 2 antibodies dominate the response to Wuhan-Hu-1 or Beta, 54% of antibodies elicited by Gamma infection recognized Class 3 epitopes. The results have implications for variant-specific vaccines and infections, suggesting that exposure to variants generally provides more limited protection to other variants.

## Teaser

This paper describes the plasma and memory antibody response in a cohort of SARS-CoV-2 Gamma-infected individuals in Brazil. Potent antibody neutralization was shown to be limited to Gamma and Beta, and epitope recognition skewed to Class 3 epitopes.

## Introduction

Over 2 yr since its onset, the coronavirus disease 2019 (COVID-19) pandemic continues to be a global problem. This is due in part to the emergence of variant strains of the SARS-CoV-2 virus. The World Health Organization has identified several variants of particular public health concern that may spread faster and/or cause more severe illness than other variants (World Health Organization, 2021). Vaccination and/or prior infection with Wuhan-Hu-1 provide different levels of protection against each of these variants, and therefore heterologous breakthrough infections are frequent, especially in individuals with waning humoral immunity (Baum et al., 2020; Betton et al., 2021; Cele et al., 2021; Choi et al., 2021; Faria et al., 2021; Garcia-Beltran et al., 2021; Greaney et al., 2021; Khoury et al., 2021; Li et al., 2020; Tao et al., 2021; Thomson et al., 2021; Wang et al., 2021b; Wang et al., 2021d; Wibmer et al., 2021; Xie et al., 2021; Zhou et al., 2021).

**Figure 1.**
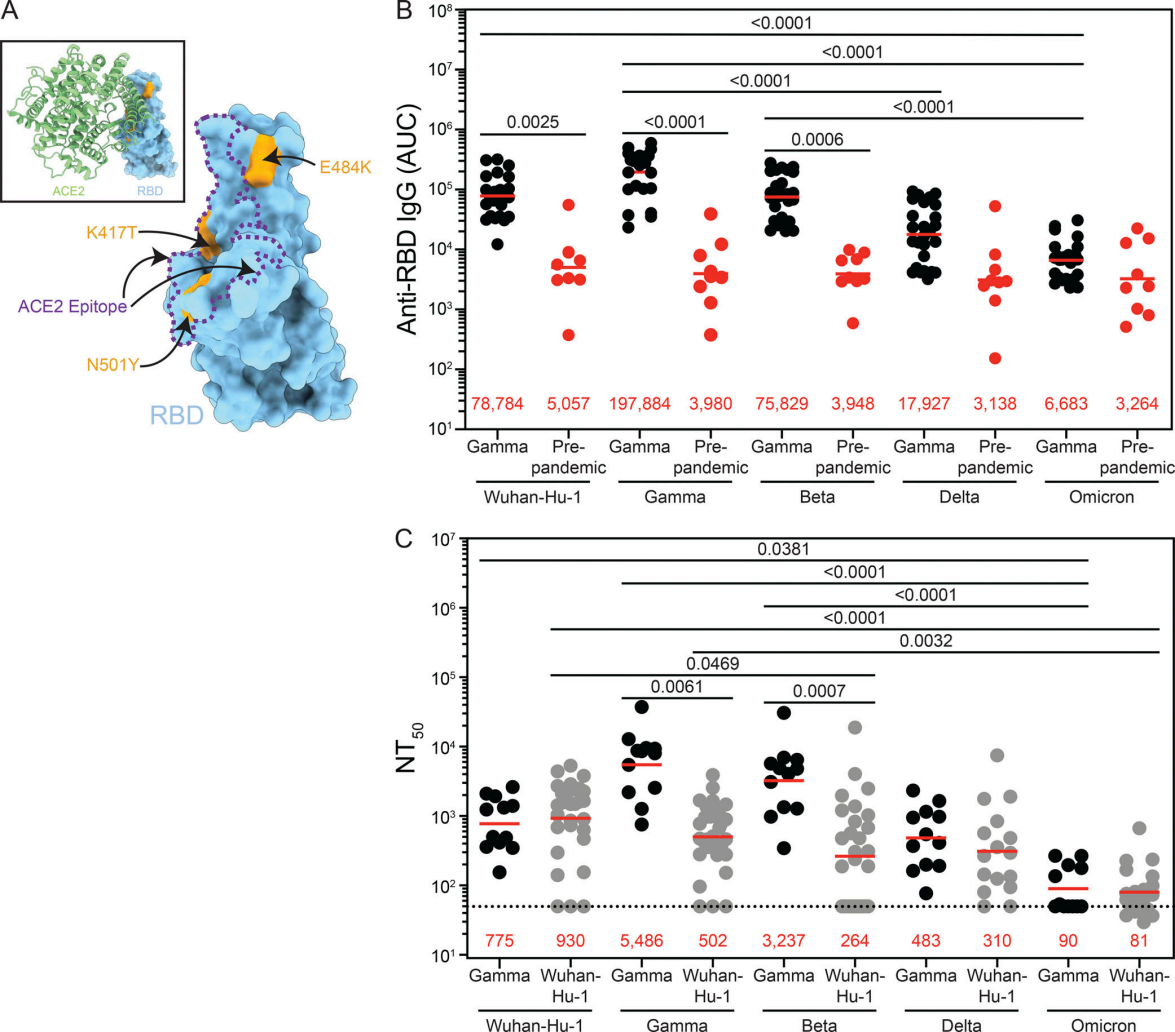
**Characterization of serological responses in Gamma-infected individuals. (A)** Surface representation of structure of SARS-CoV-2 RBD. The positions of key Gamma mutations are highlighted in orange, and the ACE-2 epitope is indicated by purple dashed lines; inset shows the ACE-2 (shown as a ribbon diagram) and RBD interaction. **(B)** Binding to Wuhan-Hu-1 and variant RBDs by plasma IgG from Gamma-infected (black) and prepandemic (red) cohorts, summarized as area under the curve (AUC). **(C)** Neutralization activity against Wuhan-Hu-1 and variant pseudoviruses by plasma IgG from Gamma-infected (black) and Wuhan-Hu-1–infected (gray) cohorts, summarized as NT_50_ values. B and C show averaged results from duplicate experiments. Numbers in red are mean geometric values; statistical differences determined by two-sided Kruskal-Wallis test with subsequent Dunn’s multiple comparisons.

The Gamma variant, or lineage P.1, was identified in Japan and Brazil in early January 2021 (da Silva et al., 2021; Faria et al., 2021; Fujino et al., 2021; Imai et al., 2021; National Institute of Infectious Diseases, 2021; Voloch et al., 2021). Gamma differs from Wuhan-Hu-1 by 17 unique amino acid substitutions, 10 of which are in the spike (S) protein. These include three critical substitutions in the receptor-binding domain (RBD) of S that alter the immunologic and biophysical properties of this domain: K417T, E484K, and N501Y (Fig. 1 A; da Silva et al., 2021; Faria et al., 2021; Voloch et al., 2021). There are also five substitutions in the N-terminal domain (NTD), namely L18F, T20N, P26S, D138Y, and R190S, of which 18F, 20N, and P26S occur in or near the supersite targeted by the majority of NTD neutralizing antibodies (Faria et al., 2021; McCallum et al., 2021).

These mutations likely arise as a result of immune pressure (Baum et al., 2020; Schmidt et al., 2021b; Weisblum et al., 2020), and result in decreased plasma neutralizing activity against Gamma as compared to Wuhan-Hu-1 in people who have been exposed to the Wuhan-Hu-1 S antigen, either in the form of vaccination or infection (Cele et al., 2021; Greaney et al., 2021; Hoffmann et al., 2021; Muecksch et al., 2021; Robbiani et al., 2020; Souza et al., 2021; Wang et al., 2021a; Wang et al., 2021c; Wang et al., 2021d). Consistent with the serological findings, the K417N/T, E484K, and N501Y substitutions found in Beta and Gamma interfere with the binding and neutralizing activity of the most abundant classes of neutralizing antibodies (Caniels et al., 2021; Dejnirattisai et al., 2021; Liu et al., 2022; Muecksch et al., 2021; Sakharkar et al., 2021; Wang et al., 2021b; Wang et al., 2021c; Wang et al., 2021d; Weisblum et al., 2020; Wibmer et al., 2021; Yuan et al., 2021).

Consistent with the antigenic distance between Beta or Gamma and Wuhan-Hu-1 or Delta viruses, infection with Beta produces plasma antibody responses that are more potent against Beta or Gamma than against Wuhan-Hu-1 or Delta (Cele et al., 2021; Moyo-Gwete et al., 2021; Reincke et al., 2022). Characterization of the antibodies produced in response to Beta revealed that they differ from those elicited by Wuhan-Hu-1 infection or vaccination because they are more focused on the K417N, E484K, and N501Y substitutions and therefore are less effective against viruses like Wuhan-Hu-1 that remain unmutated at these residues (Liu et al., 2022; Reincke et al., 2022).

Gamma differs from Beta at 13 positions including 417T/N in the RBD (Faria et al., 2021; Tegally et al., 2021). To investigate how infection with Gamma impacts antibody development, we examined the plasma and memory B cell response in individuals from the city of Manaus, Brazil that were infected with the Gamma variant (Table S1).

## Results

### Serological responses in Gamma-infected donors

We initially characterized the plasma from a cohort of 21 individuals from Manaus, Brazil hospitalized for COVID-19 infection between February and November 2021 when Gamma was the dominant variant in the Amazonas state (Faria et al., 2021). Consistent with the timing, three samples were sequenced and all three were verified to have had authentic Gamma infection. Participant ages ranged from 26 to 65 yr old (median 49 yr old) and 78% were males. Symptom onset ranged from 8 to 19 d before hospitalization (median 14 d), and documented lengths of hospitalizations ranged from 29 to 31 d. 14 of the participants (54%) were discharged after supplemental oxygen treatment. None of the participants were vaccinated (Table S1).

Plasma IgG responses to Wuhan-Hu-1 and 417N/484K/501Y RBDs were measured by ELISA (Gaebler et al., 2021; Robbiani et al., 2020). We focused on the RBD because plasma RBD antibodies strongly correlate with neutralizing activity (Brouwer et al., 2020; Cao et al., 2020; Ju et al., 2020; Robbiani et al., 2020). Plasma IgG anti-RBD binding activity against Wuhan-Hu-1, Gamma, and Beta RBDs in Gamma-infected individuals was significantly higher than pre-pandemic controls (P = 0.0025, P < 0.0001, and P = 0.0006, respectively; Fig. 1 B; Wang et al., 2021c). As expected, plasma from the Brazilian cohort binds significantly better to the Gamma RBD than Delta or Omicron RBDs (P < 0.0001 and P < 0.0001, respectively).

Plasma neutralizing activity was determined using a panel of HIV-1 pseudotyped with a panel of S proteins of SARS-CoV-2 Wuhan-Hu-1, or Gamma (P.1), Beta (B.1.351), or Delta (B.1.617), or Omicron (B.1.1.529; Gaebler et al., 2021; Robbiani et al., 2020; Schmidt et al., 2020). In contrast to the ELISAs, plasma from Gamma-infected individuals showed the highest neutralizing activity against Gamma and differing levels of activity against the other variants (Fig. 1 C and Table S1). Neutralizing activity against Wuhan-Hu-1, Beta, Delta, and Omicron was 7.1-, 1.7-, 11.4-, and 61.0-fold lower, respectively, than the activity against Gamma pseudovirus (Fig. 1 C and Table S1). Notably, Gamma-convalescent individuals show very low levels of neutralizing activity against Omicron despite three shared amino acid substitutions in the RBD of the two strains. Plasma samples obtained from Wuhan-Hu-1–infected individuals that suffered mild infections showed the highest neutralizing activity against Wuhan-Hu-1, with 1.9-, 3.5-, 3.0-, and 11.5-fold lower activity against Gamma, Beta, Delta, and Omicron pseudoviruses, respectively (Fig. 1 C).

### B cell memory response to Gamma infection

**Figure S1.**
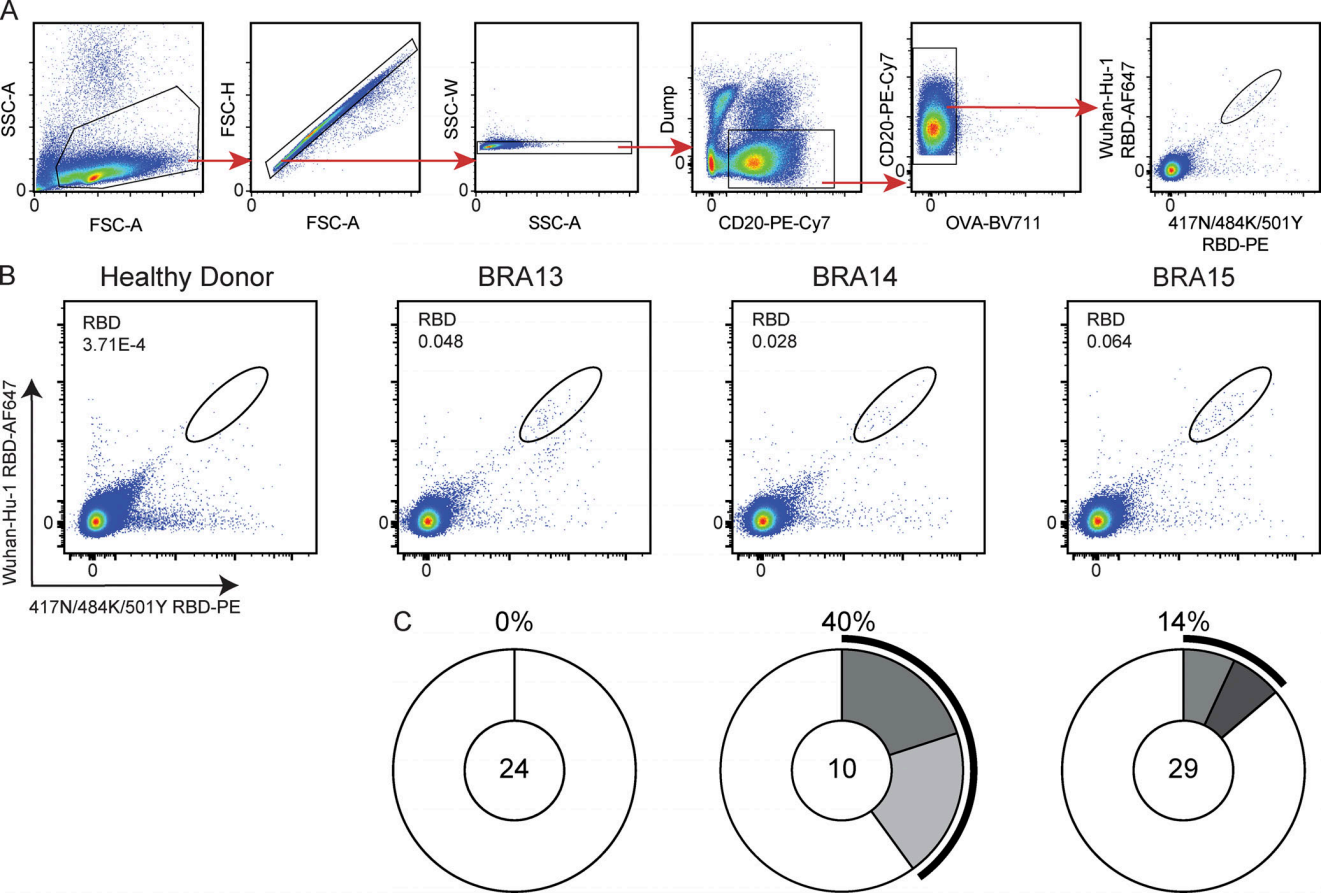
**Identification of cross-reactive anti–Wuhan-Hu-1 and anti-417N/484K/501Y RBD antibodies from Gamma-infected individuals. (A)** Gating strategy. Gating was performed on singlets that were CD20^+^ and CD3^−^ CD8^−^ CD14^−^ CD16^−^ Ova^−^. Sorted cells were Wuhan-Hu-1 RBD-PE^+^ and 417N/484K/501Y RBD-AF647^+^. **(B)** Representative flow cytometry plots showing PE-RBD– and AF-647-RBD–binding B cells for one control and three study donors. Gating strategy is shown in Fig. S2 A. **(C)** Distribution of antibody sequences obtained from three donors. The number in the inner circle indicates the number of sequences analyzed per individual. White indicates sequences isolated once, while gray slices are proportional to the number of clonally expanded sequences. The outer black arc denotes the frequency of clonal sequences per donor. Related to Fig. 2.

The memory B cell compartment contains cells that express a diverse collection of anti–SARS-CoV-2 antibodies, some of which are neutralizing (Cho et al., 2021; Robbiani et al., 2020; Wang et al., 2021c; Wang et al., 2021d). Although antibodies to the NTD and other domains of the S protein can be neutralizing, we focused on antibodies against the RBD because these are among the most potent neutralizing antibodies against SARS-CoV-2 (Kreer et al., 2020; Rogers et al., 2020). Flow cytometry was used to identify and single-cell sort circulating RBD-specific memory B cells in three Gamma-convalescent donors. To purify B cells expressing cross-reactive antibodies, we baited memory B cells using both Wuhan-Hu-1 RBD labeled with phycoerythrin and 417N/484K/501YT RBD labeled with Alexa Fluor-647 (Fig. S1 A). 24, 10, and 29 paired antibody heavy and light chain sequences were obtained from each of the three donors, respectively, for a total of 63 antibodies (Fig. S1 B and Table S2).

**Figure 2.**
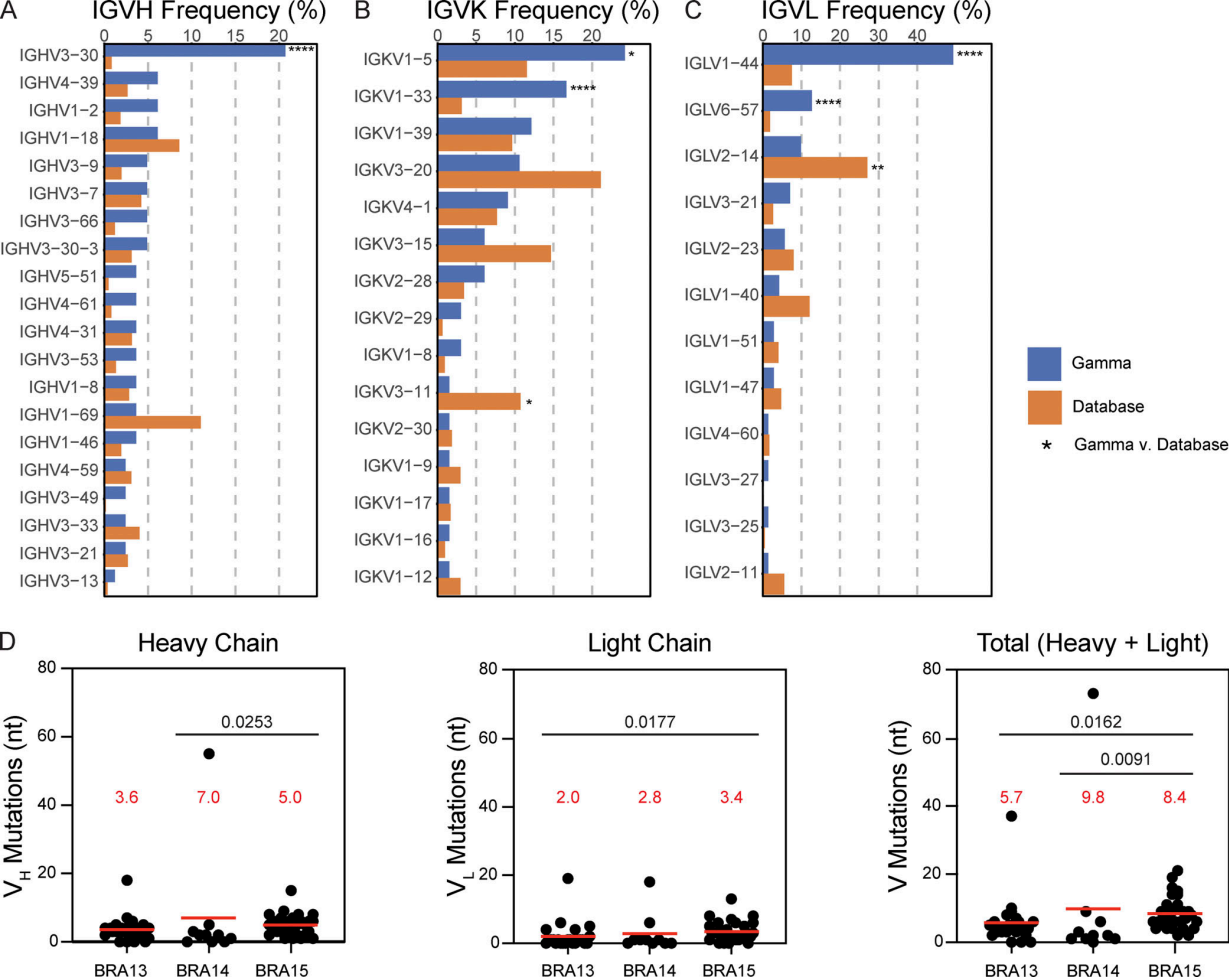
**Antibody V gene frequency and mutations. (A–C)** Bar graphs show the frequency distributions of human V genes for heavy chain (A), kappa chain (B), and lambda chain (C) in antibodies from Gamma-infected donors (blue) and Sequence Read Archive accession SRP010970 (orange). Statistical significance determined by two-sided binomial test with unequal variance; significant differences are denoted by asterisks (*, P < 0.05; **, P < 0.01; ****, P < 0.0001). **(D)** Number of somatic nucleotide mutations in IGVH (P = 0.0253 for BRA14 versus BRA15), IGVK and IGVL combined (P = 0.0177 for BRA13 versus BRA15), and total IGV (P = 0.0162 for BRA13 versus BRA15; P = 0.0091 for BRA14 versus BRA15) as indicated per donor. Red horizontal bars indicate mean values; statistical differences determined by two-sided Kruskal-Wallis test with subsequent Dunn’s multiple comparisons.

**Figure S2.**
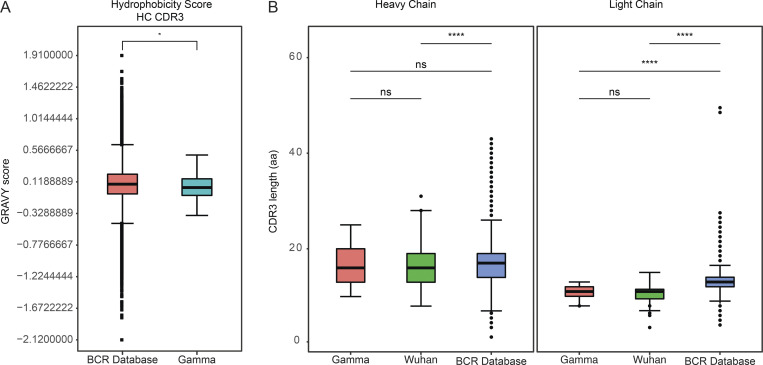
**Antibody sequence hydrophobicity and CDR3 length. (A)** Distribution of hydrophobicity GRAVY scores at the IGH CDR3 of antibodies from all donors combined and compared to human repertoire (Briney et al., 2019). **(B)** CDR3 lengths for all heavy and light chains of antibodies isolated in this study compared to human repertoire. For A and B, statistical significance is determined by two-sided binomial test with unequal variance and denoted by asterisks (*, P < 0.05; ****, P < 0.0001). Related to Fig. 2.

IGHV3-30 was significantly overrepresented in the Gamma-infected repertoire compared to the reference database, as were IGKV1-5, IGKV1-33, IGLV1-44, and IGLV6-57 (Fig. 2, A–C). The mean number of nucleotide mutations in the IGVH + IGVL genes varied from 5.7 to 8.4 (Fig. 2 D). Hydrophobicity was marginally decreased compared to the control database (Wang et al., 2021c; Fig. S2 A). Although there were no significant differences in IGH complementarity-determining region 3 (CDR3) length, light chain CDR3 length was significantly shorter than in the reference database (P < 0.0001; Fig. S2 B; Wang et al., 2021c).

### Cross-reactive but not cross-neutralizing anti-Gamma–SARS-CoV-2 antibodies

**Figure 3.**
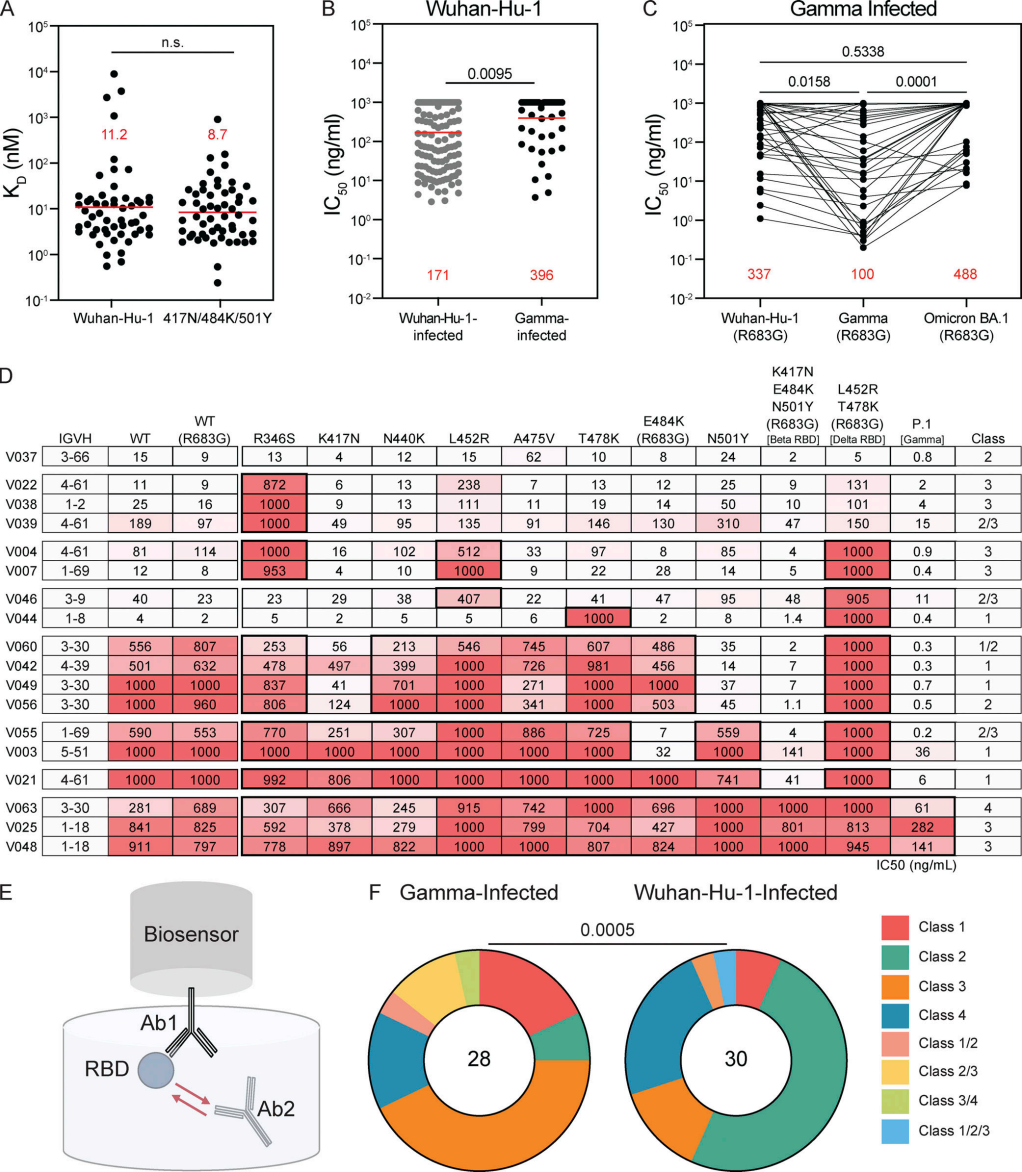
**Characterization of cross-reactive but not cross-neutralizing antibodies. (A)** K_D_ for Wuhan-Hu-1 and Gamma RBDs of antibodies from Gamma-infected cohort. Red horizontal bars indicate geometric mean values; no significant difference by two-sided Wilcoxon matched-pairs signed rank test. BLI traces shown in Fig. S2, A and B; mean K_D_ calculated based on triplicate binding curves matching theoretical fit with R^2^ value ≥ 0.8. **(B)** Neutralization of Wuhan-Hu-1 pseudovirus by monoclonal antibodies from Wuhan-Hu-1–infected (gray) and Gamma-infected (black) cohorts, summarized as IC_50_ values. P = 0.0095 by two-sided Mann-Whitney test. **(C)** Neutralization of Wuhan-Hu-1 (R683G), Gamma (R683G), and Omicron (R683G) pseudovirus by monoclonal antibodies from Gamma-infected cohort, summarized as IC_50_ values. Lines connect individual antibodies across variants. Dashed line indicates the limit of detection. P = 0.5338 for Wuhan-Hu-1 (R683G) versus Omicron BA.1 (R683G); P = 0.0158 for Wuhan-Hu-1 (R683G) versus Gamma (R683G); and P = 0.0001 for Gamma (R683G) versus Omicron BA.1 (R683G). Statistical significance determined by Friedman’s test followed by Dunn’s multiple comparisons. For A and B, red horizontal bars indicate geometric mean values. Average IC_50_ values calculated based on duplicate experiments. **(D)** IC_50_ values for *n* = 18 antibodies against indicated mutant SARS-CoV-2 pseudoviruses. Color gradient indicates IC_50_ values ranging 0 (white) to 1,000 ng/ml (red). Average IC_50_ values calculated based on duplicate experiments. **(E)** Schematic of BLI experiment. **(F)** Bar graph showing percentages of antibodies assigned to each binding class based on BLI epitope binning experiments for *n* = 28 antibodies each from Wuhan-Hu-1–infected (gray) and Gamma-infected (black) cohorts. Significance (P = 0.0005) determined using Fisher’s test for exact count data.

**Figure S3.**
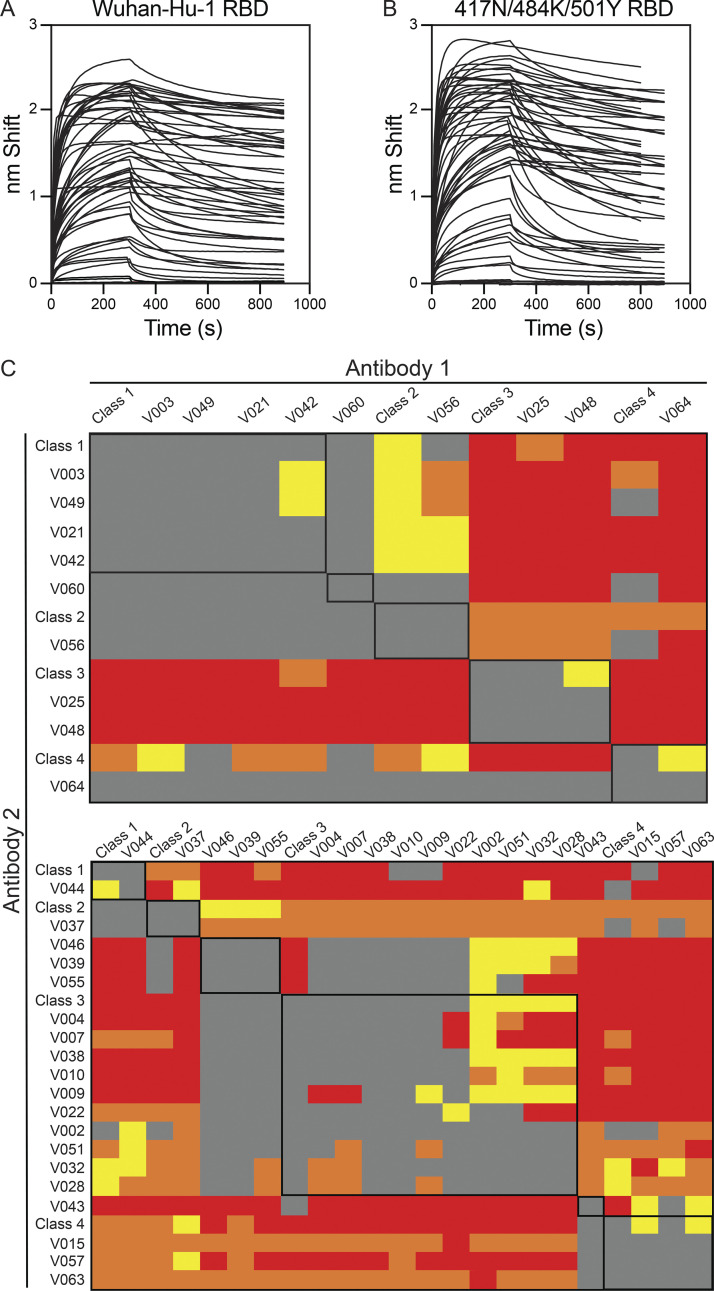
**Biolayer interferometry affinity measurements. (A and B)** Graphs depict affinity measurements of anti-Gamma monoclonal antibodies for Wuhan-Hu-1 RBD (A) or N417/484K/501Y RBD (B); data are representative of experiments performed in triplicate. **(C)** Heat maps of two biolayer interferometry competition experiments showing relative inhibition of binding of monoclonal antibody (x axis) to preformed complexes of 417N/484K/501Y RBD with another monoclonal antibody (y axis). Gray indicates no binding; yellow indicates low binding; orange, intermediate binding; and red, high binding. Data are normalized by subtraction of autologous antibody control. Related to Fig. 3.

54 antibodies were expressed and tested for reactivity to the RBD by ELISA. These antibodies include seven from expanded clones and 47 singlets (Table S3). Of the antibodies tested, 90% (49 out of 54) bound to at least one of the RBDs used as antigen bait for flow cytometry, with 89% (48 out of 54) of antibodies binding Wuhan-Hu-1 RBD and 85% (46 out of 54) of antibodies binding 417N/484K/501Y RBD. 72% (39 out of 54) bound both RBDs (Table S4). The relative affinity of antibodies as measured using biolayer interferometry (BLI) was similar for both RBDs (Fig. 3 A; and Fig. S3, A and B; and Table S4).

SARS-CoV-2 pseudotyped viruses were used to measure the neutralizing activity of the monoclonal antibodies against both Wuhan-Hu-1, Gamma, and Omicron. Of the 54 antibodies tested, 61% (33 out of 54) neutralized at least one of the three pseudoviruses (Table S4). However, geometric mean neutralizing activity was significantly worse against Wuhan-Hu-1 than observed for antibodies obtained from Wuhan-Hu-1–infected individuals (Robbiani et al., 2020; P = 0.0095, Fig. 3 B). Notably, neutralizing activity of the antibodies from the Brazilian cohort was better against Gamma than Wuhan-Hu-1 or Omicron (P = 0.0158 and P = 0.0001, respectively; Fig. 3 C). 24 of the 33 neutralizing antibodies tested showed activity against Wuhan-Hu-1 and only 13 showed neutralizing activity against Omicron, compared to 31 that neutralized Gamma (Fig. 3 C and Table S4).

To examine the breadth of these monoclonal antibodies against other variants and to further map their neutralizing epitopes, 18 of the 33 neutralizing antibodies were selected for further neutralization assays using a panel of 10 SARS-CoV-2 variant–pseudotyped viruses including Wuhan-Hu-1 (WT), R683G, R346S, K417N, N440K, L452R, A475, T478K, E484K/R683G, K417N/E484K/N501Y/R683G (Beta), L452R/T478K/R683G (Delta), and K417T/E484K/N501Y/R683G (Gamma; Fig. 3 D). Antibodies were selected to represent a range of IC_50_ values and ability to neutralize Wuhan-Hu-1 and/or Gamma, including eight strong neutralizers of both Gamma and Wuhan-Hu-1 pseudovirus (IC_50_ < 20 ng/ml and IC_50_ < 200 ng/ml, respectively); seven strong neutralizers of Gamma but poor neutralizers of Wuhan-Hu-1 (IC_50_ < 40 ng/ml and IC_50_ > 500 ng/ml, respectively); and three poor neutralizers of Gamma and Wuhan-Hu-1 pseudovirus (IC_50_ > 50 and IC_50_ > 200 ng/ml, respectively).

All 18 antibodies neutralized Gamma with IC_50_s ranging from 0.2 to 282 ng/ml with a geometric mean IC_50_ of 3.0 ng/ml, (Fig. 3 D). In contrast, only 14 and 6 of the antibodies neutralized Wuhan-Hu-1 and Delta with geometric mean IC_50_s of 159.1 and 516.7 ng/ml, respectively (Fig. 3 D). The antibodies that failed to neutralize Delta were predominantly sensitive to the individual Delta substitutions L452R and T478K, as well as a series of additional amino acid substitutions found in variants of concern. Only one antibody, V037, exhibited neutralization of all pseudotypes with a geometric mean IC_50_ of 8.5 ng/ml against all mutants (Fig. 3 D and Table S3).

To define the epitopes targeted by the neutralizing antibodies, we performed competition BLI assays in which a preformed antibody-417N/484K/501Y RBD complex was exposed to a second monoclonal antibody (schematic in Fig. 3 E). The 28 antibodies assayed were representative of the differential neutralizing activity among the strains tested, including the 18 antibodies tested for breadth (Fig. 3 C). In addition to competing against themselves, control antibodies representing the four major structurally defined classes of anti-RBD antibodies were also included: C837 (Class 1), C882 (Class 2), C908 (Class 3), and C022 (Class 1/4; Barnes et al., 2020a; Jette et al., 2021; Rogers et al., 2020; Wang et al., 2021c).

Out of the 28 antibodies tested, 12 reciprocally inhibited binding of the Class 3 control antibody, which recognizes both “up” and “down” RBDs and binds outside of the angiotensin-converting enzyme 2 (ACE-2; Robbiani et al., 2020) binding site (Fig. 3 F and Fig. S3 C; Barnes et al., 2020a). The remaining antibodies were roughly equally distributed between Classes 1, 2, and 4 (5, 3, and 5 antibodies, respectively, Fig. 3 F), although some overlap was also observed between classes (1 Class 1/2, 3 Class 2/3, and 1 Class 3/4 antibodies; Fig. 3 F). This distribution differed significantly from that seen in monoclonal antibodies isolated from the Wuhan-Hu-1–convalescent cohort (Fig. 3 F, P = 0.0005; Robbiani et al., 2020; Wang et al., 2021c), where 15 out of 30 antibodies tested reciprocally inhibited a Class 2 control antibody, which binds within the ACE-2 binding site.

## Discussion

We have examined a cohort of individuals from Manaus, Brazil hospitalized with SARS-CoV-2 infection during the peak of the Gamma variant wave in that city. As seen in previously described Wuhan-Hu-1–infected cohorts (Robbiani et al., 2020; Wang et al., 2021c), the Gamma cohort was characterized by a higher prevalence of males, and a median age of late 40s. In contrast to the other cohorts, the Gamma cohort focused on individuals hospitalized with severe disease, and so symptom severity and duration were longer than in other cohorts we studied. As shown in individuals infected with Wuhan-Hu-1, the Gamma-infected individuals developed relatively high levels of plasma neutralizing activity against the variant they were infected with (Hoffmann et al., 2021; Robbiani et al., 2020; Wang et al., 2021a; Wang et al., 2021c). The plasma neutralizing activity was higher against Gamma than against Wuhan, Delta, or Omicron, for the latter of which there was no detectable activity in 7 of the 12 individuals tested. Consistent with the relatively smaller antigenic difference between Beta and Gamma, and the relatively high activity of plasma from Beta-infected individuals against Gamma, there was only a 1.7-fold decrease in neutralizing activity against Beta (Cele et al., 2021; Moyo-Gwete et al., 2021; Reincke et al., 2022). Thus, at an early time point after Gamma infection, serologic activity focuses on the homologous isolate and closely related variants of SARS-CoV-2.

Memory B cell antibodies obtained from two cohorts of individuals infected with Beta were characterized for their binding and neutralization properties (Liu et al., 2022; Reincke et al., 2022). In both cases, the antibodies were obtained using intact S to capture antigen-specific B cells, and nearly all the potent neutralizing antibodies mapped to Class 1 or 2 epitopes. These antibodies make key contacts with mutant residues at positions 417N/484K/501Y, which helps explain why they neutralized Beta but not Wuhan-Hu-1. Similar to these previous studies, 61% of the antibodies obtained here from Gamma-infected individuals lost at least one order of magnitude neutralizing activity against Wuhan-Hu-1. However, only 45% of the antibodies obtained from Gamma-infected individuals were uniquely Class 1 or 2. This may be due to differences in the sorting strategy used to isolate antibodies. Instead of using Wuhan-Hu-1 S or RBD to capture B cells, Gamma samples were baited with a combination of Wuhan-Hu-1 and 417N/484K/501Y RBD. Nevertheless, among the Class 1 or 2 antibodies that were inactive against Wuhan-Hu-1, four out of six retained activity against viruses pseudotyped with N501Y. Thus, although Class 1 and 2 antibodies represent 45% of the potently neutralizing antibodies obtained from our cohort, the neutralizing properties of antibodies from Beta-infected cohorts in these two classes (Moyo-Gwete et al., 2021; Reincke et al., 2022) show similarities to those produced after Gamma infection.

Consistent with their reported target epitopes, only 15% of the potently neutralizing antibodies obtained from Beta-infected individuals showed decreased activity against the Delta variant (Liu et al., 2022). Notably, 83% of the antibodies obtained from the Brazilian cohort were at least one order of magnitude less active against Delta than against Gamma and 78% showed little or no measurable activity against Delta. Additionally, 76% of the antibodies isolated from the Brazilian cohort had no measurable activity against Omicron. Thus, both Beta and Gamma infection elicit memory B cells producing antibodies that are more active against these two variants; however, the antibodies obtained from individuals infected with Gamma using an RBD bait are biased to Class 3 and 4 and less active against Delta or Wuhan-Hu-1 than those obtained from Beta-infected individuals.

Of the antibodies tested against a panel of variant-pseudotyped viruses, six of the nine Class 3 Gamma-cohort antibodies exhibited broad and potent cross-neutralization, with loss of activity to R346S, L452R, and Delta RBDs. In stark contrast, only two of the eight Class 1 or 2 antibodies tested exhibited relative breadth. The data are consistent with the idea that Class 3 antibodies can be less sensitive to circulating SARS-CoV-2 variant mutations because many of the antibody escape mutations are in epitopes targeted by Class 1 and 2 antibodies (Andreano et al., 2021; Baum et al., 2020; Greaney et al., 2021; Harvey et al., 2021; Li et al., 2020; Liu et al., 2021; Weisblum et al., 2020).

Limited plasma neutralizing activity suggests that individuals that recovered from Gamma might be susceptible to subsequent infection with Delta, Omicron, and even Wuhan-Hu-1 should it reappear. In addition, if vaccination with Gamma elicits humoral and memory responses that resemble natural infection, as documented for Wuhan-Hu-1 (Wang et al., 2021d), then Gamma vaccination would be relatively less effective against other more distant variants such as Delta or Omicron. Our data are limited to a relatively short window after infection when the plasma antibody response is nearly at its peak and the memory response is evolving. Additionally, these samples represent individuals hospitalized with Gamma infection, while plasma in the Wuhan-Hu-1–infected cohort was biased to milder infections. Nevertheless, based on the plasma response in Wuhan-Hu-1–infected individuals and available data from vaccinated individuals, it would be expected that the memory response will evolve in Gamma-infected individuals (Cho et al., 2021; Gaebler et al., 2021; Wang et al., 2021c; Wang et al., 2021d). Whether breadth and potency develop further remains to be determined, but the data reported here suggests that unvaccinated sectors of the global population that have only seen one variant, such as Gamma in Brazil, remain at risk for future outbreaks with antigenically distant variants.

## Materials and methods

### Study participants

Study participants were from Manaus, transferred to the Hospital Federal do Andarai in Rio de Janeiro, and recruited through the Federal University of Rio de Janeiro in Brazil. Samples were obtained upon consent from eligible individuals, i.e., adults aged 18–65 with cases of SARS-CoV-2 Gamma infection. Variant infection was confirmed where possible during the acute phase of infection by RT-PCR. Symptoms during hospitalization and other clinical data were collected in-hospital. Peripheral blood mononuclear cell (PBMC) and plasma samples were collected 10–43 d after symptom onset. For detailed participant characteristics, see Table S1. All studies were performed in compliance with relevant ethical regulations and the protocol for studies with human participants was approved by the Institutional Review Board of The Rockefeller University.

### Blood samples processing and storage

PBMCs were obtained via gradient centrifugation using Ficoll and stored in liquid nitrogen in freezing media comprising 90% FCS and 10% DMSO. Heparinized plasma samples were stored at −20°C; prior to experiments, aliquots of plasma were heat-inactivated via incubation at 56°C for 1 h and then stored at 4°C.

### Proteins

Mammalian expression vectors encoding SARS-CoV-2 RBD (GenBank MN985325.1; S protein residues 319–539), the L452R/T478K RBD mutant, or the 417N/484K/501Y RBD mutant with an N-terminal human IL-2 or Mu phosphatase signal peptide were produced and used as previously described (Barnes et al., 2020b). Gamma (P.1) RBD and Omicron (B.1.1.529) RBD were purchased from Abbexa (cat. abx620006) and AcroBiosystems (cat. SPD-C82E4), respectively, and verified by Western blot.

### Protein biotinylation

Purified and Avi-tagged SARS-CoV-2 RBD or 417N/484K/501Y RBD mutant were biotinylated using Biotin-Protein Ligase BirA kit (Avidity) according to the manufacturer’s instructions and conjugated to streptavidin-PE (554061; BD Biosciences) and streptavidin–Alexa Fluor 647 (405237; Biolegend; Agudelo et al., 2021; Robbiani et al., 2020). Ovalbumin (A5503-1G; Sigma-Aldrich) was biotinylated using the EZ Sulfo-NHS-LC-Biotinylation kit (A39257; Thermo Fisher Scientific) according to the manufacturer’s instructions and conjugated to streptavidin-BV711 (563262; BD Biosciences; Agudelo et al., 2021; Robbiani et al., 2020). Biotinylation was confirmed by ELISA and protein-shift gel prior to use in flow cytometry.

### Single-cell sorting

As previously described (Robbiani et al., 2020), PBMCs were enriched for B cells via negative selection using a pan–B cell isolation kit (130-101-638; Miltenyi Biotec) according to the manufacturer’s protocol. Enriched B cells were incubated with fluorophore-labeled RBD and ovalbumin, and in the presence of antihuman antibodies anti-CD3-APC-eFluro 780 (47-0037-41; Invitrogen), anti-CD8-APC-eFluro 780 (47-0086-42; Invitrogen), anti-CD14-APC-eFluro 780 (47-0149-42; Invitrogen), anti-CD16-APC-eFluro 780 (47-0168-41; Invitrogen), anti-CD20-PECy7 (335793; BD Biosciences), and Zombie NIR (423105; BioLegend) in FACS buffer (1 × PBS, 2% calf serum, 1mM EDTA) for 30 min on ice. Single CD3^−^CD8^−^CD14^−^CD16^−^ZombieNIR^-^CD20^+^Ova^−^RBD^+^RBD KEN^+^ were sorted using a FACS Aria III (Becton Dickinson) into individual wells of a 96-well plate, each containing 4 μl of lysis buffer comprising 0.5× PBS, 10 mM dithiothreitol, and 3,000 U/ml RNasin Ribonuclease Inhibitors (N2615; Promega). Sorted cells were frozen on dry ice and stored at −80°C until further processing.

### Antibody sequencing, cloning, and expression

The antibody sequences obtained as described below derive from memory B cells because they originate from small CD20^+^ cells and were PCR-amplified using IgG-specific primers. RNA from lysed single cells was reverse transcribed using SuperScript III Reverse Transcriptase (18080-044; Invitrogen). cDNA from this reaction was stored at −20°C before amplification of variable Ig heavy (IGH), Ig lambda (IGL), and Ig kappa (IGK) genes by nested PCR for Sanger sequencing. The first PCR amplicons were used as template for sequence- and ligation-independent cloning into antibody expression vectors as previously described (Agudelo et al., 2021; Robbiani et al., 2020). Recombinant monoclonal IgG antibodies were produced and purified as previously described (Klein et al., 2014; Schoofs et al., 2019).

### ELISA

Binding of plasma IgG to SARS-CoV-2 proteins was measured by standard ELISA. High-binding half-area 96-well plates (3690; Corning) were coated overnight at room temperature with 50 μl per well of a 1 µg/ml protein solution in PBS. Plates were blocked with 0.1 mM EDTA, 0.05% Tween, and 2% BSA for 1 h at room temperature. After blocking, plasma diluted in PBS (1:100 starting dilution, with 10 additional threefold serial dilutions) were incubated for 1 h at room temperature. Plates were then incubated with secondary goat anti-human-IgG F(ab′)_2_ fragments conjugated to HRP (109-036-088; Jackson Immunoresearch) diluted 1:5,000 for 1 h at room temperature. Plates were developed using 3,3′,5,5′-tetramethylbenzidine substrate (34021; Thermo Fisher Scientific); the reaction was stopped after 4.5 min using 1 M H_2_SO_4_ and the plates read at 450 nm with an ELISA microplate reader (FluoStar Omega 5.11; BMG Labtech) and Omega MARS analytics software. Between each step, plates were washed six times using 0.05% Tween in PBS. A positive control (plasma from participant COV72; Robbiani et al., 2020) diluted as described was included on each plate. The average positive control’s signal was used for normalization of all other samples’ values with Excel software before calculating the area under the curve using GraphPad Prism v.9.2.0.

### Cell lines

293T cells (*Homo sapiens*; sex: female, embryonic kidney) obtained from the American Type Culture Collection (CRL-3216), 293TAce2 cells (Robbiani et al., 2020; Schmidt et al., 2020) and HT1080Ace2 cl14 cells (parental HT1080: *Homo sapiens*; sex: male, fibrosarcoma, obtained from the American Type Culture Collection [CCL-121]; Schmidt et al., 2020) were cultured in DMEM supplemented with 10% FBS at 37°C and 5% CO_2_. All cell lines have tested negative for mycoplasma contamination.

### SARS-CoV-2 pseudotyped reporter virus

A panel of plasmids expressing RBD-mutant and variant SARS-CoV-2 spike proteins in the context of pSARS-CoV-2-S_Δ19_ (Robbiani et al., 2020; Schmidt et al., 2020) has been described (Cho et al., 2021; Muecksch et al., 2021; Weisblum et al., 2020). Variant pseudoviruses resembling circulating SARS-CoV-2 variants were generated by introduction of substitutions using synthetic gene fragments (IDT) or overlap extension PCR-mediated mutagenesis and Gibson assembly (Cho et al., 2021; Schmidt et al., 2021a; Wang et al., 2021c). Specifically, the variant-specific deletions and substitutions introduced were as follows: Beta (B.1.351): D80A, D215G, L242H, R246I, K417N, E484K, N501Y, D614G, A701V; Gamma (P.1): L18F, T20N, P26S, D138Y, R190S, K417T, E484K, N501Y, D614G, H655Y, T1027I, V1167F; Delta (B.1.617.2): T19R, Δ156-158, L452R, T478K, D614G, P681R, D950N; Omicron (B.1.1.529): A67V, Δ69-70, T95I, G142D, Δ143-145, Δ211, L212I, ins214EPE, G339D, S371L, S373P, S375F, K417N, N440K, G446S, S477N, T478K, E484A, Q493K, G496S, Q498R, N501Y, Y505H, T547K, D614G, H655Y, H679K, P681H, N764K, D796Y, N856K, Q954H, N969H, N969K, L981F.

The L452R/T478K substitution, as well as the deletions/substitutions corresponding to variants listed above, was incorporated into a spike protein that also includes the R683G substitution, which disrupts the furin cleavage site and increases particle infectivity. Neutralizing activity against mutant pseudoviruses was compared to a WT SARS-CoV-2 spike sequence (Genbank NC_045512), carrying R683G where appropriate.

SARS-CoV-2 pseudotyped particles were generated as previously described (Robbiani et al., 2020; Schmidt et al., 2020). Briefly, 293T (CRL-11268) cells were transfected with pNL4-3ΔEnv-nanoluc and pSARS-CoV-2-S_Δ19_, and particles were harvested 48 h after transfection, filtered, and stored at −80°C.

### Pseudotyped virus neutralization assay

Fourfold serially diluted prepandemic negative control plasma from COVID-19–convalescent individuals or monoclonal antibodies were incubated with SARS-CoV-2 pseudotyped virus for 1 h at 37°C. The mixture was subsequently incubated with 293T_Ace2_ cells (Robbiani et al., 2020; Schmidt et al., 2020; for all WT neutralization assays) or HT1080Ace2 cl14 (Schmidt et al., 2020; for all mutant panel and variant neutralization assays) cells for 48 h, after which cells were washed with PBS and lysed with Luciferase Cell Culture Lysis 5 × reagent (Promega). Nanoluc Luciferase activity in lysates was measured using the Nano-Glo Luciferase Assay System (Promega) with the Glomax Navigator (Promega). The relative luminescence units were normalized to those derived from cells infected with SARS-CoV-2 pseudotyped virus in the absence of plasma or monoclonal antibodies. The half-maximal neutralization titers for plasma (NT_50_) or half-maximal concentrations for monoclonal antibodies (IC_50_) were determined using four-parameter nonlinear regression (least-squares regression method without weighting; constraints: top = 1, bottom = 0; GraphPad Prism).

### BLI

BLI assays were performed as previously described (Robbiani et al., 2020; Wang et al., 2021c). Briefly, the Octet Red instrument (ForteBio) was used at 30°C with shaking at 1,000 rpm and protein A biosensors (18-5010; ForteBio). Kinetic analysis was performed as follows: (1) Baseline: 60 s immersion in buffer; (2) Loading: 200 s immersion in buffer; (3) Baseline: 200 s immersion in buffer; (4) Association: 300 s immersion in WT or N417/484K/501Y RBD diluted to 100, 50, 25, 20, 10, or 5 µg/ml in buffer; (5) Dissociation: 900 s immersion in buffer. Affinity measurements were corrected by subtracting signal obtained for each IgG in the absence of WT or N417/484K/501Y RBD as appropriate. Curve fitting was performed with a fast 1:1 binding model and Fortebio Octet Data analysis software (Fortebio). Mean dissociation constants (K_D_) values were calculated from the average of all binding curves matching theoretical fit with R^2^ value ≥0.8. Epitope-binding assays were performed as follows: according to the manufacturer’s protocol for “Classical Sandwich Assay”: (1) Sensor Check: 30 s immersion in buffer; (2) Capture first antibody: 10 min immersion in Ab1 diluted to 30 µg/ml in buffer; (3) Baseline: 200 s immersion in buffer; (4) Blocking: 5 min immersion in IgG isotype control diluted to 50 µg/ml in buffer; (5) Antigen association: 300 s immersion in N417/484K/501Y RBD diluted to 100 µg/ml in buffer; (6) Baseline: 30 immersion in buffer; (7) Association second antibody: 5 min immersion in Ab2 diluted to 30 µg/ml in buffer. Curve fitting was performed using Fortebio Octet Data analysis software (Fortebio).

### Statistical analysis

Data were analyzed using Wilcoxon nonparametric tests, two-sided Mann-Whitney tests, or two-sided Kruskal-Wallis and Friedman’s tests with Dunn’s multiple comparisons as specified, calculated in GraphPad Prism (version 9.2.0, GraphPad Software). Fisher’s test for exact count data performed using the https://astatsa.com/FisherTest online calculator. P values <0.05 were considered significant.

### Computational analysis of antibody sequences

Antibody sequences were trimmed on the basis of quality and annotated using Igblastn v.1.14 with the IMGT domain delineation system. Annotation was performed systematically using Change-O toolkit v.0.4.540. Heavy and light chains derived from the same cell were paired, and clonotypes were assigned on the basis of their V and J genes using in-house R and Perl scripts. All scripts and the data used to process antibody sequences are publicly available on GitHub (https://github.com/stratust/igpipeline/tree/igpipeline2_timepoint_v2). The antibody sequences have been deposited into GenBank (accession numbers: ON703911–ON704035).

The frequency distributions of human V genes in anti–SARS-CoV-2 antibodies from this study were compared with 131,284,220 IGH and IGL sequences generated in (Soto et al., 2019) and downloaded from cAb-Rep (Guo et al., 2019), a database of shared human B cell antigen receptor clonotypes available at https://cab-rep.c2b2.columbia.edu/. On the basis of the 52 distinct V genes that made up the 63 analyzed sequences from the Ig repertoire of the three participants present in this study, we selected the IGH and IGL sequences from the database that were partially encoded by the same V genes and counted them according to the constant region. The frequencies shown in Fig. 2 are relative to the source and isotype analyzed. We used the two-sided binomial test to check whether the number of sequences belonging to a specific IGHV or IGLV gene in the repertoire was different according to the frequency of the same IGV gene in the database. Adjusted P values were calculated using the false discovery rate correction. Significant differences are denoted with asterisks.

Nucleotide somatic hypermutation and CDR3 length were determined using in-house R and Perl scripts. For somatic hypermutations, IGHV and IGLV nucleotide sequences were aligned against the closest germline sequences using Igblastn and the number of differences was considered to correspond to nucleotide mutations. The average number of mutations for V genes was calculated by dividing the sum of all nucleotide mutations across all participants by the number of sequences used for the analysis.

### Online supplemental material

Fig. S1 shows the single-cell sorting strategy and pie charts describing the clonality of the antibodies expressed by memory B cells sorted using the described strategy. Fig. S2 shows hydrophobicity GRAVY (grand average of hydropathy) scores and CDR3 length analysis for Gamma-convalescent donors compared to the general human B cell receptor repertoire. Fig. S3 includes affinity measurements for antibodies to Wuhan-Hu-1 RBD and 417N/484K/501Y RBD, and heat maps describing antibody epitope binning on 417N/484K/501Y RBD. Table S1 contains clinical and demographic data for Gamma-convalescent donors. Table S2 includes all antibody sequences obtained from sorted donor samples. Table S3 contains the sequences of all monoclonal antibodies cloned and recombinantly expressed. Table S4 shows EC_50_, K_D_, and IC_50_ values for all antibodies tested.

## Supplementary Material

Table S1 shows cohort characteristics.

Table S2 includes all antibody sequences obtained from sorted donor samples.

Table S3 shows sequences of cloned recombinant monoclonal antibodies.

Table S4 contains effective and inhibitory concentrations of monoclonal antibodies.
